# The Foreign Journals: Surgery

**Published:** 1841-01

**Authors:** 


*Gazette Médicale de Paris. Septembre* 5, 1840.

### SURGERY.

*On Itch and its Treatment.* By Dr. DE LA HARPE, Chief Physician of the Hospital of Lausanne.

THE author gives the following formula for an ointment which he has employed in upwards of 400 patients. It does not appear to irritate the skin, and is said to be better than the liniment of Valentin or the alkaline sulphur ointment of Alibert. The mean duration of treatment was eighteen days in 1836, fifteen in 1837, eleven in 1838, and ten in 1839. The formula is as follows:

| | |
|---|---|
| Flowers of sulphur - - - | 16 parts. |
| Sulphate of zinc - - - - | 2 „ |
| Powder of white hellebore - | 4 „ |
| Soft soap - - - - - - - | 31 „ |
| Lard - - - - - - - - | 62 „ |

*Gazette Médicale. Juillet*, 1840.

---

*On the Use of Mercurial Plaster ("Emplâtre de Vigo") in preventing Deformity after Smallpox.* By M. CHOMEL.

A young girl is under M. Chomel, in the Hôtel Dieu, who has been vaccinated, but she says without effect. The variolous eruption, which is demiconfluent, was preceded by the ordinary symptoms. A mask, made with the plaster of Vigo, was applied to the face on the second day of the eruption. The patient tore it off within twenty-four hours, but, notwithstanding this, the result is very remarkable. On the neck, chest, and all the rest of the body, the variolous pustules were developed with all their distinguishing characters: opaque, umbilicated, and surrounded by a bright red areola. But on the face the course has been very different; instead of pustules we see acuminated vesicles, or solid papulæ. On some points where the plaster had not adhered, small pustules are perceived ; where it had, we search for them in vain. It appears impossible, on the simple inspection of this patient, to state that mercury has not exercised a local action, specific and advantageous ; for the patient will not be scarred, and will be speedily convalescent.

In another ward is a patient already convalescent, who has been submitted to the same mercurial treatment, and the desquamation has followed its ordinary course, except on the face, where scales have not formed. In another bed in the same ward is a woman five months and a half advanced in pregnancy, in whom the plaster has entirely averted the eruption from the face : small whitish papulæ supplying the place of the pustules. Desquamation has not taken place, except on the lips and eyelids, where some pustules appeared ; but the mask of plaster did not extend to these parts. The treatment is well worthy of further trial.

*Bulletin Général de Thérapeutique.* 15 *et* 30 *Août*, 1840.

---

*On the Employment of large Doses of Tartarised Antimony in the Treatment of Articular Dropsies.* By M. GIMELLE.

M. GIMELLE, who some time ago published a series of facts, illustrative of the utility of tartar emetic in hydarthrosis, has continued his observations, and finds that when an articulation is the seat of synovial effusion, the same treatment is the most effectual for procuring speedy resorption. In twenty-eight cases the emetic tartar was given in increasing doses, commencing with four grains in the twenty-four hours, and increasing two grains daily till the dose was sixteeen, eighteen, or twenty grains per diem, with the invariable effect of determining the resorption of the liquid in a space of time, varying from eight to sixteen days. Of twenty-eight cases of effusion of synovia into articulations, twenty-two had their seat in the knee-joint ; three were double, and two were in the shoulder-joint ; one was in the elbow, and one in the ancle. All the patients took the emetic tartar in infusion of linden tree with syrup of poppies. In eighteen the tolerance was established on the first day, in two on the second, and in two on the third. No accident occurred to any of the patients after the tolerance was established. The dose of twenty grains was never exceeded, and in all the cases the effusion was absorbed in the space of eight, ten, or sixteen days—longest period during which the remedy was administered. In twenty-five cases the pain and stiffness felt in the affected articulations diminished at the same time and degree with the effusion, and when the latter had disappeared, the patients could walk as easily as before they were attacked by the disease. In two cases, however, though the liquid disappeared in the ordinary time, the pain remained in one case for a month and in the other for forty days. In one case the remedy was carried to twelve grains without benefit, and as it was one of very old standing, it was thought proper to relinquish the medicine.

We give the following cases, as they are very interesting proofs of the value of the treatment employed.

CASE I. A man, aged seventy-three, was affected by a very large hydarthrosis of the left knee, which extended into the hollow of the ham, where it formed a tumour of the size of the fist, which disappeared on strong pressure, and became visible on each side of the patella. M. Pasquier prescribed the emetic after the form of M. Gimelle. The dose was successively carried to 16 grains ; the patient experienced no inconvenience, his appetite continued good, and on the sixteenth day all the signs of effusion into the articulation had disappeared. Two days afterwards the patient was dismissed entirely cured.

CASE II. On the 10th of September M. Gimelle was called to a student, aged twenty-one years, with an hydarthrosis of the right knee, which had been treated without success during six weeks by leeches, blisters, compression, frictions, and embrocations. M. Gimelle prescribed the tartar emetic. The patient's appetite continued good, and he had no inconvenience. The dose of twelve grains was not exceeded, and fourteen days after this treatment was commenced there was no trace of synovial effusion, the patient feeling merely a feebleness of the limb.

Case iii. A healthy female, aged twenty-three, in a journey from Tours to Paris, caught cold during the night, and, on her arrival in the capital, experienced pains in the right knee. M. Gimelle considered this case one of commencing hydarthrosis, and prescribed without delay a gummy potion with four grains of tartar emetic and an ounce of syrup of poppies. Five or six vomitings, and afterwards alvine dejections followed, but the pain was relieved in the night and the patient could move the limb. On the second day the catamenia appeared, and the emetic was suspended for five days, during which the pain reappeared and the effusion increased, and the articulation became very red and hot. The emetic was then resumed, four grains being given on the first and second day; it produced three or four vomitings, and as many stools. Tolerance was established on the second day, there was a diminution of the synovial tension, and the patient could bear slight movements of the limb without much pain. On the third day six grains were given, eight on the fourth, ten on the fifth, twelve on the sixth, and fourteen on the seventh; the tolerance continued, and the amelioration was progressive. On the eighth, ninth, and tenth days, sixteen grains were given, and on the tenth day the synovial effusion had completely disappeared.

In none of these cases did M. Gimelle precede the employment of the tartar emetic by local or general bloodletting. Nevertheless, he thinks that if the fever be intense, and the articulation present great heat and redness, or if there be great irritation of the digestive organs, it would be proper to combat these symptoms before administering the emetic tartar. By this preliminary treatment we should diminish the chances of gastric irritation, probably facilitate the tolerance, and consequently the action of the remedy.

The most constant effects of the tartar emetic were diminution in the force and rapidity of the pulse, enfeebling of the voice, fatigue and coloration of the eyelids (known by the name of *yeux cérnes*), and abundant perspirations during the night. Five patients had vomitings, two during one day, one two days, and two during three days. Eight had very abundant alvine dejections, lasting from one to three days: in three the vomiting and purging coexisted. Sixteen experienced neither vomiting nor purging. In the majority the appetite was unaltered; and in those cases where it was disturbed, it was reestablished with the tolerance. The quantity of urine was diminished, which M. Gimelle attributed to the abundant perspirations. All the other functions were performed as in the healthy state. The quantity of food the patients had taken when in health was not diminished, and often increased. Lastly, M. Gimelle saw all the patients some months after treatment, and many of them after some years, and in none did any accident occur.

*Bulletin Général de Thérapeutique.* 15 *et* 30 *Juillet,* 1840.

---

### *Case of immense Cæcal Fistula in the Lumbar Region.*
### By Dr. Meding, of Meissen.

A strong man, aged thirty-six, suffered for some days from griping sensations in the abdomen; on the sixth day I found him with the most complete symptoms of a severe psoitis of the right side, which I believed was the result of either rheumatism from cold, or of a sprain from over-exertion. General and local abstractions of blood, several times repeated, mitigated the pain, but produced no essential change in the state of the patient; a constant restlessness prevented him from sleeping; his pulse was accelerated, but full; his breathing anxious; his tongue moist and furred; he was thirsty and had lost his appetite; his skin was alternately cold and hot; his bowels very loose; his urine depositing an abundant red sediment.

The severe pains in the back from the right lumbar region to the shoulder, and forwards to the testis and the thigh, as well as the great tenderness strictly limited to the right iliac and inguinal region, suggested an affection of the cæcum. But although as the disease went on, there was every reason to believe that effusion or suppuration must have taken place, yet no part could be found at which the matter might be evacuated. Only on the back, the common belly of the sacro-lumbalis and longissimus dorsi appeared somewhat prominent, though without any alteration in the colour or elasticity of the skin. At this part however the author determined to make an exploratory incision, and on the 24th day of the disease he cut along the outer edge of the common belly of the muscles just mentioned for four inches upwards from the crista ilii, through the skin and a thick layer of fat down to the external oblique. All these tissues were perfectly healthy; but on introducing a lancet an inch deep into the fibres of the abdominal muscles, a little pure pus gushed out with the blood, and was soon followed by a stream of blackish-gray ichor of a most fetid macerated-bone-like smell. For several days the discharge continued to be very profuse, and the patient became extremely weak. Every day there came away with the acrid irritating ichor masses of cellular tissue, partly from the interstices of the outer muscles of the back, and partly from the fat and cellular substance surrounding the kidney and psoas muscle. The finger could now be easily passed over the transverse processes to the anterior surface of the bodies of the lumbar vertebræ, and the psoas and iliacus muscles could be distinctly felt quite cleared of cellular tissue. Such examinations always excited the severest cramps of the chest, and twitchings of the right lower extremity. With a long probe it was found that on the inner side the whole lumbar region of the abdomen behind the peritoneum from the kidney into the cavity of the pelvis, and on the outer side the interspaces of the dorsal muscles from the inferior angle of the right scapula to the middle of the outer surface of the ilium, formed one cavity, the source of the putrid fluid.

For six weeks after the operation the condition of the patient seemed hopeless; he became weaker and more emaciated, had severe spasms of the pharynx and rectum, and was in a state of utter prostration. At the end of this time, however, and quite unexpectedly, the sloughing away of the cellular tissue gradually ceased, the wound assumed a more healthy aspect, the matter discharged became thicker and more purulent, acquired a stercorous odour, and soon after was found to contain small portions of fæces. At the same time the general condition of the patient improved.

The whole of the cavity above described was filled twice a-day with dry charpie pushed into it as far as possible with a long and thick piece of whalebone. On passing the latter as a probe forwards and downwards over the crista ilii, one came in the neighbourhood of the fifth lumbar transverse process, immediately at the outer edge of the quadratus lumborum, to an aperture as big as a large quill, through which the whalebone passed to a depth of six or eight inches, till it was stopped by a soft yielding substance. There could be no doubt that this hole led into the cæcum; and it was found that by closing it accurately with charpie, and keeping the patient on his left side, with a careful diet and open bowels, the evacuation of fecal matter through the wound was entirely prevented. With some unimportant interruptions, the gradual closure of the cavity went on steadily after the eighth week from the operation. The quantity of charpie necessary to fill it up (which had at first been very considerable) decreased with the diminution of the discharge; the thigh which had been fixed in the flexed posture became more and more moveable; and all the functions of the body were gradually restored to their natural state. In rather more than ten months from the day on which the cavity was opened, the external wound was completely healed; no relapse took place; the muscles which had been implicated in the disease gradually gained power, and in rather less than two years from the commencement of his malady, the patient was restored to his former robust health.

*Von Ammon's Monatsschrift für Medicin, u. a. Juli,* 1840.

*Subcutaneous Section of Forty-two Muscles, Tendons, or Ligaments, practised the same day, on the same person, to remedy a general articular deformity.* By M. JULES GUERIN.

IN a letter addressed to the Academy of Sciences on the 31st of August, 1840, M. Guérin states that on the 25th of that month he performed the section of forty-two tendons, muscles, or ligaments, to remedy a series of articular deformities of the trunk and limbs, caused by the active retraction of these muscles and ligaments. There were twenty-eight openings of the skin required. The following were the parts divided:

| | | |
|---|---|---|
| Trunk | Pectoralis major | 1 |
| The arms | On each side biceps cubiti | 2 |
| | „ pronator teres | 2 |
| | „ extensor carpi radialis | 2 |
| | „ flexor communis sublimis | 2 |
| | „ palmaris brevis | 2 |
| The forearms | Tendons of extensor carpi ulnaris on each side | 2 |
| | „ palmaris longus and brevis | 4 |
| | „ abductor pollicis | 2 |
| The legs | The sartorius on each side | 2 |
| | The biceps cruris | 2 |
| | The semi-membranosus | 2 |
| | The semi-tendinosus | 2 |
| | The rectus femoris | 2 |
| | Fascia lata | 1 |
| | External lateral ligaments of knee | 2 |
| The feet | The tendo Achillis on each side | 2 |
| | The tibialis anticus | 2 |
| | The extensor communis | 2 |
| | The extensor proprius pollicis | 2 |
| | The peronei antici | 2 |
| | | 42 |

The patieut only experienced moderate pain or fatigue, and did not complain during the operations, which lasted an hour. An hour afterwards he was in a sound sleep. He was very tranquil the following night and day. No inflammatory accident supervened, and on the third day, the twenty-eight wounds were completely cicatrized. The sections were made in the presence of many distinguished French and foreign surgeons. M Guérin holds that he is not open to the charge of rashness, as he first established the absolute innocuousness of subcutaneous wounds by numerous experiments on animals, and then verified the same principle in man by a series of operations from the section of one muscle to that of a great number. He purposes to lay an account of his method of operating with its definite results before the Academy in a future memoir.

*Gazette Médicale de Paris. Septembre* 5, 1840.

---

*On a New Species of Torticollis.* By M. BOUVIER.

THIS species of torticollis differs altogether ftom the acute or chronic muscular form, and from the spontaneous luxation of the atlas and axis, three states with which it has been confounded. It has its seat in the articulations of the first cervical vertebræ, and may perhaps be designated by the term articular torticollis. The lateral articulations, right or left, of the first cervical vertebræ, and especially of the atlas with the axis, are, so to speak, the focus of articular torticollis, which consists in a peculiar form of inflammation of the synovial capsule and fibrous tissues of these articulations. The essential characters are: 1. A position of the head and neck, similar to that produced by contraction of one of the sterno-cleido-mastoid muscles, namely, lateral flexion to the right or left, and rotation of the face to the opposite side. 2. A pain felt towards the top and sides of the nape of the neck, sometimes on the lateral and posterior parts of the cranium; arising spontaneously, or in the movements of the neck. 3. Complete relaxation of the sterno-cleido-mastoid of the affected side, and an equal tension of the muscles of the neck both left and right. 4. Stiffness and pain in all or any of its movements, notwithstanding the preservation of the articular connexions of the cervical vertebræ, and the integrity of the muscles. 5. Absence of swelling, and of sensibility on pressure. 6. Atrophy, or arrest of development of the side of the face, which answers to the inclination of the head, when this has lasted a certain time.

This disease presents two successive states or periods, the acute and chronic. It is often produced by cold, sometimes by sudden distension of the ligaments. M. Bouvier thinks it very important to detect this disease, as the section of the muscle would be useless and rash, but reserves to another occasion the proceedings which he has found useful.

*L'Expérience. 17 Septembre*, 1840.

---

*Wound of the Urinary Bladder.* By Dr. SCHÜTTE, of Mullheim.

A healthy man, thirty-seven years old, fell perpendicularly from a height of about eight feet, on an upright wooden stake several feet long and fully an inch thick. Its end passed into the inner surface of the left thigh, about three inches from the rectum, and ran into the lower part of the urinary bladder above the sphincter vesicæ. The urine flowed continually and insensibly through the wound; but neither blood nor urine passed through the urethra. A catheter was placed in the urethra, and several leeches and lotions of cold water were applied externally; and when the danger of severe inflammation had passed by, bread and water poultices were put over the wound. The patient was allowed only mild food, and in about three weeks the wound had healed without any ill consequences.

*Medicinische Zeitung. October* 7, 1840.

---

*On the Treatment of Fissures of the Anus by Rhatany.* By M. TROUSSEAU

A mode of treatment successful in these troublesome cases without the necessity of the knife or cauterization has long been desired. We therefore publish the results M. Trousseau has obtained by the employment of rhatany. M. Bretonneau first introduced this treatment, and the numerous cures effected by him induced M. Trousseau to employ it, with the effect of saving the patient from a very painful if not dangerous operation. This means is one which appears scarcely rational, as it is well known that spasmodic constrictions of the sphincter play an important part in this disease, and rhatany injected into the rectum is *a priori* one of the most likely means to augment this constriction. To this M. Trousseau replies, that it matters little whether the remedy be irrational or not, provided it cure the patient. During the month of January, 1839, five patients were thus treated, of whom four were cured. MM. Marjolin, Bérard, and Desquibses, have also each cured a patient by the rhatany. Some of the cases are given at length, but we do not think it necessary to detail them.

With regard to the modus operandi of the remedy, M. Trousseau enquires, Do the tannin and gallic acid, so abundant in extract of rhatany, the action of which is so powerfully astringent, force away the blood which accumulates towards the irritated part, and by thus dissipating the inflammatory congestion favour rapid cicatrization? Or does the additional tonicity imparted by this medicine to the sphincter muscles, to the mucous membrane, and the submucous cellular tissue, enable the tissues more effectually to resist the distension caused by the passage of the mass of excrement; so that the wound, no longer enlarged each day, will naturally tend to cicatrize? However this be, M. Trousseau thinks that there is some special property in rhatany which cures fissures of the anus as quinine cures fever, and mercury and iodine syphilis. He believes that all vegetable substances approaching rhatany in their chemical composition would have the same therapeutic effects, and this belief is strengthened by the success MM. Payer and Manec have obtained by the topical application of "monesia," a remedy recently imported into France, which among other ingredients contains a remarkable quantity of tannin.

The mode of employing the rhatany appears very simple, and the following is the method recommended by M. Trousseau, with slight alterations to suit individual cases. He gives the patient an enema, either of olive or almond oil,or of mucilage, and half an hour afterwards another containing five ounces of water, a drachm to a drachm and a half of extract of rhatany, and half a drachm of alcohol. The patient must retain this, and have a similar one at night. When the acute pain is allayed he is only to have one lavement a day, and when the cure appears to be completed one every two days for a fortnight. He has tried without advantage suppositories composed of a drachm and a half of cocoa-nut oil, and from eighteen to thirty-six grains of rhatany; but it would appear that "mèches" covered with an ointment composed of one part of rhatany and six or eight of lard or cerate, are useful in some cases.

*Bulletin Général de Thérapeutique.* 15 *et* 30 *Août*, 1840.

---

*On the Use of Boiling Water in the Treatment of Callous Fistulæ.*
By Dr. Ruppius, of Freiburg.

The author was induced to adopt this method of treatment from what he had seen it effect in the hands of Rust of Vienna, and from the observation that the granulations which grow from scalded parts of the skin are peculiarly florid, and prone to unite firmly; a consideration which, we may add, long ago induced French surgeons to adopt the actual cautery for the same means, and is the foundation of the very skilful operations of M. Lallemand for vaginal fistulæ. Two cases are related, one of recto-vaginal fistula from abscess after a severe labour; the other, of an incomplete fistula in ano, extending four inches up the side of the rectum. The treatment consists in introducing the pipe of a syringe filled with boiling hot water down to the further end of the fistula, (which, if necessary, must be closed there with a finger of the other hand,) and slowly injecting a part of the contents. At the first operation only so much of the water should be forced in as is sufficient to stimulate the end of the fistula, so that it may commence healing at its deepest part, and, after the repeated injections, may make gradual progress in healing towards the surface. In both the cases that are related this procedure was strikingly successful.

*Fricke and Oppenheim's Zeitschrift. Juli,* 1840.

---

*On a New Method of Operating for the radical Cure of Hernia.*
By M. Velpeau.

The author of this memoir, after examining at length the various means hitherto employed for the cure of hernia, states that he finds none of them at all efficacious; he therefore determined to try another method. Convinced from repeated experiments that injections with tincture of iodine produced in the serous membranes an adhesive inflammation, slight and scarcely dangerous, he attempted the radical cure of hernia by these injections. Three patients were operated on by him in the years 1836 and 1837, but the difficulties of a first operation, and the vagueness of the results, still left him in doubt, and he afterwards employed a method in two cases consisting of three distinct steps: subcutaneous puncture, scarification of the interior of the sac, and compression. He took the idea of scarifications from the ancients, or rather from what he had himself advanced in the year 1832. Twice that year he had placed great confidence in them, and only discontinued their employment when he perceived that danger was likely to ensue to the patient from opening the sac. These dangers seemed no longer likely, when M. Guérin gave the hint of the possibility of penetrating to the bottom of the cavity, by a simple puncture of the integuments, and expressed in his memoir his desire of applying his subcutaneous incisions to the radical cure of hernia. M. Velpeau has performed the necessary scarifications in this manner. With regard to the compression, he applies it not only to the external abdominal ring, but over the internal ring, and the inguinal canal itself. He employs the celebrated bandages of M. Fournier of Lempdes, which he states have more than once sufficed for the radical cure of hernia.

M. Velpeau successfully operated on a patient in the following manner: Having introduced the index-finger for some lines in depth into the external abdominal ring, he pushed the integuments before it, and passed a sort of lancet over the nail of this finger, pushing it obliquely as far as the iliac fossa; then withdrawing his finger from the ring, he brought the edge of the instrument against the iliac walls of the abdomen, which he supported with the other hand, and then scarified them in various directions, taking especial care to avoid the epigastric vessels. The lancet was then withdrawn at the exact spot where it had entered, and the operation was complete, only a few drops of blood having been lost. The patient was taken to bed, no accident occurred, the small wound cicatrized by the next day, the bandage of Fournier was applied on the third day, and the man has since walked and conducted himself as if he had never been afflicted.

Encouraged by this result, the patient demanded a similar operation on another hernia. It was performed, and at the end of ten days he was convalescent. He has since continued quite well, and has remained as a cook in the hospital to show the radical cure to the profession.

*Bulletin Général de Thérapeutique.* 15 *et* 30 *Août,* 1840.

---

*Solution and Absorption of Provisional Callus during Typhus Fever.*
By Dr. SCHILLING.

AN artilleryman received a fracture of the left femur on the 1st of September, and by careful management the ends of the bone were so firmly united in the middle of November, that he could bear some weight on the foot. Symptoms of typhus abdominalis, however, then set in, and ten days afterwards callus could no longer be felt, and the bones moved as freely and as easily upon one another as at the first examination after the reception of the injury. In six days more the patient died. The examination exhibited no trace of callus; the broken surfaces were bloody, like those in a recent fracture, and were surrounded by a sac-like membrane, which contained some black bloody fluid.

*Medicinische Zeitung. September* 16, 1840.

